# Measuring research impact in Australia’s medical research institutes: a scoping literature review of the objectives for and an assessment of the capabilities of research impact assessment frameworks

**DOI:** 10.1186/s12961-017-0180-1

**Published:** 2017-03-21

**Authors:** Simon Deeming, Andrew Searles, Penny Reeves, Michael Nilsson

**Affiliations:** 1 Hunter Medical Research Institute, Lot 1, Kookaburra Circuit, New Lambton Heights, Newcastle, 2305 NSW Australia; 2 School of Medicine and Public Health, The University of Newcastle, University Drive, Callaghan, Newcastle, 2308 NSW Australia; 3 Department of Medicine, John Hunter Hospital, Hunter New England Local Health District, Kookaburra Circuit, New Lambton Heights, Newcastle, 2305 NSW Australia

**Keywords:** Research impact assessment frameworks, Objectives, Productivity, Medical research, Health research, Speed of translation, Prospective orientation

## Abstract

**Background:**

Realising the economic potential of research institutions, including medical research institutes, represents a policy imperative for many Organisation for Economic Co-operation and Development nations. The assessment of research impact has consequently drawn increasing attention. Research impact assessment frameworks (RIAFs) provide a structure to assess research translation, but minimal research has examined whether alternative RIAFs realise the intended policy outcomes. This paper examines the objectives presented for RIAFs in light of economic imperatives to justify ongoing support for health and medical research investment, leverage productivity via commercialisation and outcome–efficiency gains in health systems, and ensure that translation and impact considerations are embedded into the research process.

This paper sought to list the stated objectives for RIAFs, to identify existing frameworks and to evaluate whether the identified frameworks possessed the capabilities necessary to address the specified objectives.

**Methods:**

A scoping review of the literature to identify objectives specified for RIAFs, inform upon descriptive criteria for each objective and identify existing RIAFs. Criteria were derived for each objective. The capability for the existing RIAFs to realise the alternative objectives was evaluated based upon these criteria.

**Results:**

The collated objectives for RIAFs included accountability (top-down), transparency/accountability (bottom-up), advocacy, steering, value for money, management/learning and feedback/allocation, prospective orientation, and speed of translation. Of the 25 RIAFs identified, most satisfied objectives such as accountability and advocacy, which are largely sufficient for the first economic imperative to justify research investment. The frameworks primarily designed to optimise the speed of translation or enable the prospective orientation of research possessed qualities most likely to optimise the productive outcomes from research. However, the results show that few frameworks met the criteria for these objectives.

**Conclusion:**

It is imperative that the objective(s) for an assessment framework are explicit and that RIAFs are designed to realise these objectives. If the objectives include the capability to pro-actively drive productive research impacts, the potential for prospective orientation and a focus upon the speed of translation merits prioritisation. Frameworks designed to optimise research translation and impact, rather than simply assess impact, offer greater promise to contribute to the economic imperatives compelling their implementation.

## Background

The economic potential of research institutions, including medical research institutes (MRIs), remains high on the policy agenda for many Organisation for Economic Co-operation and Development (OECD) nations [1–4]. As a consequence, the question of how to measure and assess returns from investment in health and medical research (HMR) remains a highly policy-relevant issue. Research impact assessment frameworks (RIAFs) provide a conceptual framework and methods against which the translation and impact of HMR can be assessed. Reviews by the Canadian Academy of Health Sciences (CAHS) [5], Brutscher [6], Banzi and Moja [7], and Milat and Bauman [8] summarise in detail the form and characteristics of existing RIAFs. However, a review of this literature demonstrates that, despite the centrality of purpose in evaluation [9], the objective of assessment is rarely instructive of the form, method and content of these RIAFs.

Impact assessment of HMR research expenditure has been elevated on the policy agenda through three inter-related economic imperatives:

1. Scarcity – Largely as a result of stimulatory fiscal policy during the global financial crisis, general government debt as a percentage of gross domestic product through 2014, remained markedly higher than pre-crisis levels for the majority of OECD nations, including Australia [10]. The accompanying political pressure to manage this debt, while simultaneously stimulating economic growth, heightens the justification necessary for on-going public expenditure in HMR, against competing budget priorities.
2. Productivity – Health service budgets have been cut or frozen substantially in many OECD nations, including Australia, intensifying the demand for innovative research solutions that improve health outcomes with the same or less public expenditure [11]. In the commercial realm, economic growth is increasingly reliant upon a knowledge-intensive industry, and policymakers are keen to optimise commercial innovations in medical services, pharmaceuticals and medical devices [12]. Citing Australia’s strong research capability, the McKeon Strategic Review of Health and Medical Research [13] identified potential productivity gains in both commercial and non-commercial arenas founded upon opportunities to leverage HMR and its supporting ecology. As a consequence, the requirement to generate economic productivity via greater commercial returns and cost-effective improvements to health outcomes represents a second imperative for impact assessment.
3. Efficacious, effective and cost-effective research outcomes – The third economic imperative arises from within HMR. An increasing level of research on HMR has highlighted numerous areas of unproductive expenditure via practices ranging from poorly-informed research questions and weak study design to irreproducible outcomes and failed implementation [14–17]. Chalmers and Bracken [14] estimate that, globally, $240 billion of annual biomedical research expenditure is wasted. Their research illustrates the potential to improve productive health outcomes by ensuring that translation is embodied throughout the research process.

We contend that these imperatives necessitate prioritisation of a different set of objectives, with according implications for the impact assessment of Australia’s MRIs.

Australia supports approximately 70 independent MRIs, which collectively facilitate the investigations of approximately 10,100 researchers [18]. The Institutes’ relationship with researchers varies from direct employment to facilitation of research conducted by employees of affiliated organisations, such as local health districts, universities or the private sector. The breadth of HMR conducted within Australia’s MRIs is extensive, ranging across populations, disease, policy, practice and medical products [19], and from basic science through to public health. The Institutes receive funding through competitive grants, fellowships and programs, state government infrastructure programs, grants from foundations and trusts, and commercial returns from intellectual property, contracted research and community donations. In addition to preferential taxation status, government support comprises approximately half of this funding [18]. To justify this support from government and the community, Australia’s MRIs continue to progress their approach to impact assessment. This research seeks to inform upon the development of assessment frameworks for MRIs.

The first aim of this paper was to list the stated objectives for RIAFs. The second aim sought to identify existing frameworks. The final aim sought to evaluate whether the identified frameworks possessed the capabilities necessary to address the specified objectives.

The definitions adopted for this paper include:

- Research activity – Activities necessary to conduct medical and health research, where research is defined as “*creative work undertaken on a systematic basis in order to increase the stock of knowledge, including knowledge of man, culture and society, and the use of this stock of knowledge to devise new applications*” [20].
- End-users – Agents along the pathway to impact that utilise the research outputs, including members of the community, the public sector, industry and other researchers. As such, this definition includes both interim and final users.
- Research outputs –The knowledge deliverables produced by research activity, e.g. peer-reviewed papers, presentations, contributions to collaborative endeavours, guidelines, education, prototypes, etc.
- Research outcomes/Interim impacts – Research outputs transform to research outcomes/interim impacts following utilisation by an end-user along the pathway to final impact.
- Final impacts – The demonstrable effect from the flows of knowledge between basic, patient and population-orientated research, and clinical trials, that improves human health and quality of life, and generates benefits for society, the economy, culture, national security, public policy, or the environment [21].

## Methods

**Fig. 1:**
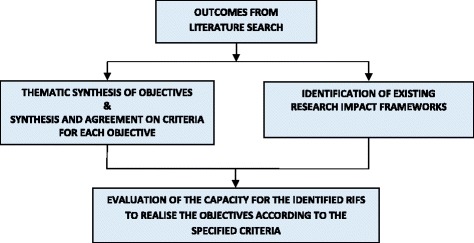
Flow diagram – Methods

**Fig. 2:**
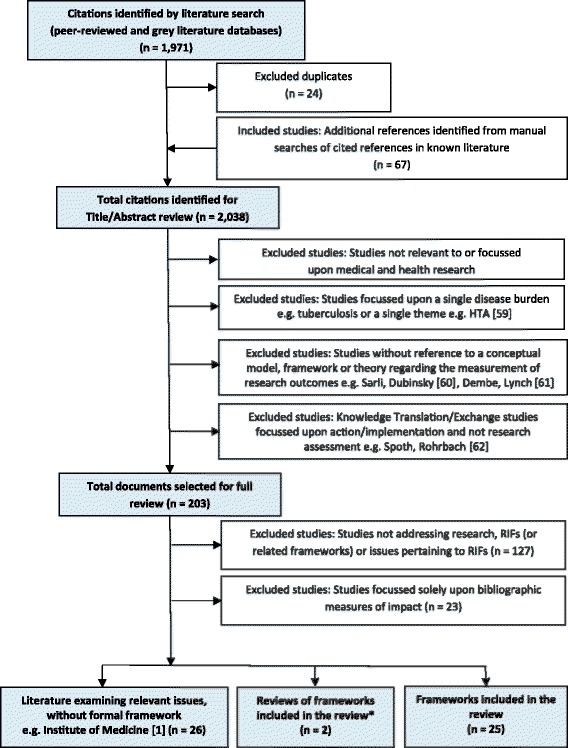
Flow diagram – Literature selection protocol [59–62]. *Note: Excludes reviews presented prior to an included framework

The methods for this study are summarised in Fig. 1. We undertook a scoping review of the literature [22]. The review comprised a systematic search of a finite number of key publication databases, a rigorous review of the grey literature and extensive hand searching of cited references [23, 24] (Fig. 2).

The literature search strategy included a search of peer-reviewed academic literature held within Scopus and grey literature referenced within OpenGrey, WHOLIS (Who Database), WorldWideScience.gov and Science.gov. The databases were searched using the following strategy:

- KEY ((translation* W/1 (research OR knowledge)) OR “knowledge mobili?ation*” OR “research into practice” OR “translation to health application*” OR “translation to patient*” OR “translation to health practice*” OR “translation to population health impact” OR “research impact” OR “knowledge into practice” OR “populari?ation of research” OR “research generated knowledge”) AND
- TITLE-ABS-KEY (metric* OR framework* OR payback OR “social return” OR sroi OR measure* OR “financial return*” OR “political impact” OR “policy impact*” OR “Social impact*” OR bibliometrics OR econometrics OR “economic evaluation*” OR “cost effectiveness” OR “cost benefit analysis”)

The search was limited to studies published in English from January 2005 to June 2015. Studies not focussed upon medical or health research were excluded. Only papers that referred to theory, a conceptual model or framework pertaining to the evaluation of research outcomes were included. Studies that solely examined bibliographic impact were excluded. Frameworks to encourage best practice through guidelines/checklists and assessments focussed upon a singular medicine or health research area without reference to the utility of the framework were also excluded.

The results of the search strategy and the exclusion criteria are summarised in Fig. 2. In summary:

- An initial review of abstracts against the inclusion criteria in the databases of academic publications found 1971 abstracts.
- Searches of reference lists and the grey literature identified a further 67 documents.
- The review of full papers and documents identified two reviews, 25 frameworks and 26 documents containing relevant information.
- A number of frameworks represented minor variations upon an established framework and were excluded. For example, the Banzi and Moja [7] framework was considered similar to the original CAHS [5] framework, and that of Rubio and Schoenbaum [25] similar to the Trochim and Kane [26] framework for the purpose of this analysis.

The first aim was realised through a thematic synthesis of the literature to identify specified objectives and common criteria. The objectives were identified independently by two researchers. Criteria for each objective were derived and independently reviewed by a second researcher. The existing RIAFs were also identified from the literature by two researchers. For this purpose, a RIAF was defined as a conceptual model/framework and/or a collection of evidence designed to assess the translation or impact of HMR beyond traditional academic outcomes.

The second aim was realised through identification of existing research impact assessment frameworks from the literature search. The final aim was realised through an evaluation of the respective capabilities of the identified frameworks. Two researchers independently assessed the capacity of each RIAF to meet the alternative objectives based upon the descriptive criteria. The evaluation results were categorised according to the following exclusive definitions:

- Yes/No – The RIAF could/could not meet the criteria specified for each respective objective;
- Partial – The RIAF could meet the criteria for the respective objectives, but was not relevant to the full spectrum of HMR; or
- Possible – The RIAF is capable of meeting the specified criteria for the respective objectives with modest adaptions

Given that the evaluators possessed significant knowledge regarding RIAFs and that guesswork was unlikely, a percentage agreement statistic was used to test inter-rater reliability. Following the initial review, inconsistencies were discussed and a mutually-agreed outcome determined. Unresolved differences are presented in the results.

## Results

### Objectives for RIAFs

The thematic analysis of the literature identified eight categories under which the objectives for a RIAF could be grouped (Table 1), namely accountability (top-down); transparency/accountability (bottom-up); advocacy; steering; value for money; management/learning and feedback/allocation; prospective orientation; and speed of translation. Accountability for the consumption of resources and the consequences of HMR represents the most common objective for impact assessment [6]. However, the question of ‘to whom is the research accountable’ generates different implications. A RIAF that provides accountability for aggregate HMR expenditure on a broad national or sectoral scale, may provide sufficient information for government or national funders, but insufficient information for accountability by individual institutions or research programs. An objective to provide transparency [1] is closely aligned with accountability at a more granular level. Transparency to research activity, outputs and outcome steps along a pathway to impact provides accountability for these steps and consequently, the potential to improve outcomes. Hence, for the purpose of the subsequent evaluation, these objectives were separated into accountability (top-down) and transparency/accountability (bottom-up) with according criteria.

**Table 1:** Categories of objectives identified for research impact assessment frameworks (RIAFs)

Objective	Review criteria/criterion for RIAFs
Accountability (top-down)	Provides information that could be utilised to provide accountability for research impact at an aggregated national, state or sector level for government, funding bodies or community stakeholders
Transparency/Accountability (bottom-up)	1. Provides transparency to the research activity, outputs and outcome steps along the pathway to impact 2. Provides information that could be utilised to provide accountability for research at smaller units of aggregation, e.g. research projects, research programs, individual institutes
Advocacy	Provides a demonstration of capability for relevant stakeholders
Steering	Capacity to align the research agenda towards a specific target, e.g. improving the diagnosis, treatment and care of people with dementia
Value for money	The outcomes enable broader comparison through standardisation into Quality-Adjusted Life Years, Disability-Adjusted Life Years, other comparable health outcomes, monetised benefits, employment or similar
Management/Learning and Feedback/Allocation	Capacity for the information produced by the RIAF to inform subsequent strategic management decisions within or between research organisations, potentially including fund allocation
Prospective orientation	1. Capacity for a framework to be implemented prospectively 2. Compels prior determination of the potential final impact and the pathway to this goal 3. Incorporates process metrics that allow for prior scrutiny of the anticipated steps towards impact
Speed of translation	1. Compels prior determination of the potential final impact and the pathway to this goal 2. Contains process metrics that enable individual actors (researchers, teams) to be accountable for steps on the pathway towards final impact 3. Includes a logic model component that necessitates ‘use’ for an outcome to be acknowledged

Advocacy was also commonly presented as an objective for research assessment [5]. While the audience for advocacy includes politicians, funders, policymakers, philanthropists, researchers and the wider community, the implications for RIAFs remain largely consistent. Consequently, the criterion adopted for advocacy reflects the ability of a RIAF to demonstrate capability to relevant stakeholders.

The capacity for a framework to steer research to address specific HMR goals represents a less common objective [6, 27]. All impact frameworks steer research towards societal benefits, but not all are designed to align the research agenda towards a specific target, e.g. improving the diagnosis, treatment and care of people with dementia. For example, a framework that solely utilises a case study methodology is ill-placed to realise this objective [28]. In the subsequent evaluation, the capacity for a framework to align the research agenda to a specific target represents the criterion for a Steering objective.

Value for money is commonly inferred as an objective for research assessment, but rarely raised explicitly in the literature [29, 30]. It is possible to define value for money as the proportion of non-monetised outcomes to inputs (resources consumed). However, if the information from a RIAF seeks to encourage productivity, broader comparison through standardisation into utility measures, such as Quality-Adjusted Life Years, other comparable health outcomes, monetised benefits or similar would be necessary [31]. The criterion for the evaluation reflects this requirement.

Learning and feedback represents a common objective in the literature, while general management was occasionally specified. Funding allocation represents another related, but less common objective [32]. Given that financial allocation is dependent upon sufficient management/learning and feedback information, these three objectives were collated. The criterion determined for the evaluation reflected the capacity for the information produced by the RIAF to inform subsequent strategic management decisions, such as fund allocation, within or between research organisations.

The final two objectives are less commonly identified in the literature. Evaluating the United States National Institute of Health Clinical and Translational Science Awards funding, Trochim and Kane [26] specify the length of time for research translation as a definitive objective. An objective to improve the speed of translation seeks to expedite health improvements and closely aligns with the economic rationale of rates of return, in this case from HMR investment. To realise improvements in the length of time for translation, the assessment framework requires three elements. Firstly, identification of the intended final health impact and where the research lies along the pathway to this potential impact. Secondly, the inclusion of process metrics that enable individual actors (researchers, teams) to be accountable for steps along the pathway to final impact. Thirdly, the inclusion of a logic model component that necessitates ‘use’ for an outcome to be acknowledged as generating an interim impact. The criteria to assess whether a framework has the capacity to pro-actively improve the speed of translation are subsequently determined by these qualities.

The objective of prospective orientation of research is explicitly raised by the Hunter Medical Research Institute Framework to Assess the Impact from Translational Health Research (HMRI FAIT) framework [21] as prospective implementation supports the use of performance monitoring and feedback. This objective is closely aligned with the speed of translation, as productive adjustments can be made prior to resource expenditure [21]. However, these themes have been kept discrete as an objective to improve the speed of translation may imply requirements beyond prospective implementation and vice versa. The criteria for this latter objective reflected the capacity for a framework to be implemented prospectively, whether it compelled prior determination of the anticipated final impact and whether it allowed for prior scrutiny of the anticipated steps towards this goal via process metrics.

### Identification of existing RIAFs

The review identified 25 RIAFs that met the literature inclusion criteria (Table 2). While inclusion was narrowly focussed upon frameworks pertaining to the evaluation of research outcomes, the focus, conceptual form and methods utilised varied markedly. The breadth of methods utilised by these frameworks include pure metrics, such as the engagement and impact framework proposed by Australian Academy of Technology and Engineering [33], pure economic models [34], and mixed methods utilised by CAHS [5], through to the peer-reviewed case studies of the United Kingdom’s Research Excellence Framework (REF) [28, 35]. Some frameworks are focussed upon particular aspects of translation, such as that of Landry and Amara [36], while others seek to address the research process across the translation spectrum [26, 37]. Collected reviews of the form and content of these frameworks have been published elsewhere [6–8, 38].

**Table 2:** Capacity for existing research impact assessment frameworks to address specific objectives

Conceptual framework	Exemplar/s	Relevant research spectrum^a^	Primary methods	Accountability (top-down)	Account. (bottom-up)/transparency	Advocacy	Steering	Value for money	Management/learning & feedback	Speed of translation	Prospective orientation of research
Balanced Scorecard	Young and Lindquist [42]	T1–T4	Quant. indicators	Partial	Yes	Partial	Yes	Possible	Yes	Possible	Possible
Canadian Academy of Health Sciences (CAHS) Impact Framework	CAHS [5]	T1–T4	Mixed Methods	Yes	Yes	Yes	No	Yes	Yes	No	Possible
Canadian Institutes of Health Research (CIHR) Impact Framework	Bernstein, Hicks [29]	T1–T4	Quant. Indicators, case studies	Yes	Yes	Yes	Possible	Yes	Yes/possible	No	Possible
Comprehensive Research Metrics Logic Model	Engel-Cox, Van Houten [55]	T3, T4	Quant. Indicators	Yes/possible	Yes	Yes	Possible	Yes	Yes	No	Possible
Decision Making Impact Model	Lavis, Ross [27]	T3, T4	Mixed methods	Yes	Yes	Yes	Possible	No/partial	Yes	Partial/possible	Yes
Economic Impact Assessment	Deloitte [41], Glover, Buxton [34]	T1–T4	Economic assessment	Yes	No	Yes	No	Yes	Partial	No	No/possible
Excellence in Research for Australia	ARC [50]	T1–T4	Peer-review, Quant. Indicators	Partial	No	Partial	No	No	Partial	No	No
Health Services Research Impact Framework	Buykx, Humphreys [51]	T1–T4	Quant. Indicators, survey	Yes	Yes	Yes	No/possible	Partial	Yes/partial	No	Possible
Hunter Medical Research Institute Framework to Assess the Impact from Translational Health Research	Searles [21]	T1–T4	Mixed Methods	Yes	Yes	Yes	Possible	Yes	Yes	Yes	Yes
Institute for Translational Health Sciences Kellogg Logic Model – World Health Organization Health Services Assessment Model	Scott, Nagasawa [47]	T1–T4	Mixed Methods	Yes	Yes	Yes	Yes/possible	Partial	Yes	No	Possible
Lean/Six-sigma Models	Schweikhart and Dembe [43]	T1–T4	Quantitative Indicators	Partial	Yes	No	Possible/no	No	Partial	Yes/partial	Yes
Matrix Scoring System	Wiegers, Houser [53]	T1–T4	Quant. Indicators	Yes	Yes/partial	Yes	No	No	Yes	No	No
Measurement of Research Impact and Achievement	National Health & Medical Research Council [48] (Not implemented)	T1–T4	Quant. Indicators, Self-report (externally verifiable)	Yes	Yes	Yes	Yes	Yes	Yes	No	Yes/possible
Payback Model of Health Research Benefits	Buxton and Hanney [45]	T1–T4	Mixed methods	Yes	Yes	Yes	No	Possible	Yes	No	Possible
Process Marker Model	Trochim, Kane [26]	T1–T4	Quant. Indicators	No	Yes	Possible	No	Possible	Yes	Yes	Possible
RE-AIM Model	Glasgow, Vogt [54]	T4	Quant. Indicators	Partial/yes	Yes	Yes	No	Partial	Yes	Partial	Possible
Research Engagement for Australia	Australian Academy of Technology and Engineering [33]	T2, T3, T4	Quant. Indicators	Partial	No	Partial	No	Partial	Partial	No	No
Research Excellence Framework	Ovseiko, Oancea [28], Khazragui and Hudson [35]	T1–T4	Quant. Indicators, survey	Yes	No	Yes	No	Possible	Yes/possible	No	Possible
Research Impact Framework	Kuruvilla [40]	T2, T3, T4	Self-assessed survey	No	Yes	Yes	No	No/partial	Yes	No	No
Research Performance Evaluation Framework	Schapper, Dwyer [32]	T1–T4	Quant. Indicators, self-assessed survey	Yes	Yes	Yes	Yes	Partial	Yes	Possible	Possible
Research Utilization Ladder	Landry, Amara [36]	T3, T4	Self-assessed survey	No	Partial	No	Possible	No	Partial/no	Partial/no	Possible
Societal Impact Framework	Mostert, Ellenbroek [52], Van Ark G [58]	T3, T4	Quant. Indicators	Partial	Yes	Yes	Possible	No	Yes	No	Possible
Telethon Kids Institute Research Impact Framework	Telethon Kids Institute [46]	T1–T4	Mixed methods	Yes	Yes	Yes	Possible	Yes	Yes	Possible	Possible
Translational Research Organizations Performance Model	Pozen and Kline [44]	T1–T4	Quant. Indicators	Yes	Yes	Yes	Possible	Partial	Yes	Yes	Yes
Weiss Logic Model	Weiss [49]	T1–T4	Mixed methods	Yes	Yes	Yes	Possible	Yes	Yes	Possible	Possible

^a^Utilises the Khoury, Gwinn [37] definition of T1–T4

Yes/No – Met/Did not meet specified criteria; Partial – Meets criteria, but incomplete representation; Possible – With adaptions, capable of meeting criteria

### Framework evaluation

This study sought to evaluate the capacity of the identified frameworks to realise the criteria specified for each objective; the results of this evaluation are summarised in Table 2. The initial percentage agreement statistic for the classification of each framework measured 62.5% between the two independent researchers. Following discussion of the differences and reclassification, the agreement statistic measured 92%. Where differences remain unresolved, both responses are tabulated. Differences largely reflected moderate variations in the interpretation of the capabilities of the conceptual models. Note that some frameworks are capable of realising objectives beyond those specified in their development.

#### Accountability (top-down)

The majority of existing RIAFs met the criteria to provide information that could be utilised to provide holistic (top-down) accountability for government, funding bodies or community stakeholders. Frameworks classified as ‘partial’ provided few aggregations, e.g. Six-sigma/lean model, or limited breadth, e.g. Research Utilization Framework, the Australian Research Council’s Excellence in Research Australia (ARC ERA) impact measures.^1^ The form of this information varies across peer-reviewed qualitative studies of impact [36, 39, 40], exclusively quantitative analyses [26, 33, 34, 41–44] and numerous mixed method assessments [5, 21, 27, 45–47].

#### Transparency/accountability (bottom-up)

Under this criterion, at least 18 frameworks provide sufficient transparency to determine a level of (bottom-up) accountability to a range of stakeholders. Frameworks, such as the Economic Impact Analysis (EIA), provide no transparency below the national or sectoral scale of analysis and consequently cannot incentivise research activity [41]. The Australian Academy of Technology and Engineering’s Research Engagement for Australia framework provides no transparency to non-commercial/non-financial impacts. It was consequently categorised as ‘partial’, given the limited incentive provided to drive economic productivity within the public health or not-for-profit sectors [33].

#### Advocacy

Nineteen of the frameworks reviewed provide information suitable for advocacy and a further three frameworks are partially suitable. Only the Balanced Scorecard and Six-sigma/lean models are potentially limited, due to their focus upon performance indicators at the expense of aggregate capability. Frameworks that incorporate a case study method potentially have an advantage for advocacy purposes. The narrative can assist the comprehension of specialist users (policymakers, clinicians), as well as non-specialists (politicians, funding bodies, philanthropists, the community), and potentially captures impacts missed by other methods [7]. Case studies are, however, limited for other objectives by selection, conceptualisation and reporting bias [40].

#### Steering

Steering the research agenda was explicitly raised as an objective for one framework [27] and one of the reviews [6]. All impact frameworks steer research, directly or indirectly, towards societal benefits. Whether a framework could align the research agenda towards a specific target, e.g. improving the diagnosis, treatment and care of people with dementia, represented the criteria for assessment. The Measurement of Research Impact and Achievement (MORIA) framework^2^ provides a prospective assessment, where the research proposal could be designed and prospectively assessed against the realisation of the targets for tied funds [48]. The focus upon outcomes within the Weiss framework also suggests that a logic model approach could be readily adapted to this objective [49]. In contrast, some frameworks, such as ARC ERA, EIA and the REF, are designed for retrospective analysis and consequently lend themselves poorly to steering [28, 35, 41, 50]. Finally, a number of the frameworks could accommodate the pre-specification of a health ‘target’ [21, 46, 51, 52]. For example, the Decision Making Impact Model explicitly considers the source of the demand for research knowledge, from which impact assessment through the ‘user-pull’ method could be conducted [27].

#### Value for money

The capacity for the assessment frameworks to realise the value for money objective is mixed. Only eight frameworks definitively have the capacity to provide information to assess value for money. Most of these frameworks are relatively broad in scope and, consequently, include methods that could provide value for money assessments, e.g. economic assessment. It is assumed for this assessment that macroeconomic impact assessments [34, 41] only provide for international or disease burden comparison and are consequently classified as partial for the purpose of a value for money objective. Nearly half of the frameworks were categorised as ‘partial’ or ‘possible’, alluding to two factors. Firstly, there is a lack of clarity regarding the purpose and therefore for the definition of value for money. Secondly, ambiguities often remain with the question of ‘value for money to whom?’.

#### Management, learning, and feedback and allocation

The majority of frameworks met this criteria, albeit that the emphasis varies from the relative impact of research outputs [5, 44, 45, 51] to organisational aims, such as progress towards strategic goals [32, 53]. While the methodologies adopted within the frameworks varied, most provide the grounds to facilitate comparison, albeit that greater transparency assists comparison in the “*doing, diffusion and impact of research within and across research fields*” [40]. A number of the frameworks were utilised as the managerial basis for disbursement of research funds [1, 32, 39, 53].

#### Speed of translation

When evaluated against the specified criteria, very few frameworks are designed to optimise the speed of translation (Table 2). The Process Marker Model, the Research Utilisation Ladder and the Decision Making Impact Model were designed with the pathway to impact and end-users in mind [26, 27, 36]. The latter two are, however, limited by their focus solely upon final end-users of research. If imperatives, such as the lag to final impact [5, 28, 44] and sub-optimal research practices within HMR [14–17], are to be addressed, then a framework requires a broader interpretation of steps in the process of research translation. The Balanced Scorecard represents another performance management framework that provides for steps along the pathway to impact, but fails to guide the critical link to usage at each step [42]. The Lean/Six-sigma models incorporate ‘customer’ needs, but favour technical and precisely quantifiable systems. The Reach Effectiveness Adoption Implementation Maintenance (RE-AIM) model was developed for public health programs [54]. Consequently, while logic model components relating to the adoption, implementation and maintenance of research interventions emphasise usage, RE-AIM does not readily extend to similar aspirations for biomedical, clinical efficacy or health systems research. The University of Washington Institute for Translational Health Sciences Kellogg Logic Model – World Health Organization Health Services Assessment (ITHS KLM–WHO) model, fulfils the criteria, but the emphasis upon outputs and a passive approach to feedback does not provide for the pro-active acceleration of translation [47]. The characteristics of HMRI FAIT, the Process Marker Model and Translational Research Organizations Performance Model appear most capable of realising this objective. While limited in some respects, the Decision Making Impact Model, Lean/Six-sigma, RE-AIM and the Research Utilization Ladder also possess qualities that are informative for the realisation of this objective.

#### Prospective orientation of research

Similar to the speed of translation, the capacity for frameworks to realise an objective to prospectively orientate research is mixed (Table 2). Inflexible retrospective formats disqualify the existing ARC ERA, EIA, Matrix Scoring System and the Research Engagement for Australia [29, 33, 41, 50, 53]. The Health Services Research Impact Framework and the Research Impact Framework were designed partly to guide researchers in the prospective planning of research implementation [40, 51], but provide limited guidance regarding process metrics that align research to anticipated impacts. MORIA was developed with a consideration to ‘drive desirable behaviours’ [48]. The intended implementation of this framework at the grant review stage provides for the prior orientation of research to optimise productive research outcomes. However, MORIA was focussed upon the track record of the researcher, as opposed to the research process. Consequently, the specified metrics do not provide flexibility to tailor interim targets (process metrics) in line with individual pathways to impact. This stated, the track record provides an incentive for researchers to realise potential impacts.

Prospective implementation of the HMRI FAIT framework on a number of research programs, including National Health and Medical Research Council Centres of Research Excellence, has successfully contributed to shaping research activity regarding the purpose, focus, viability, intended outcomes and, potentially, the research methods adopted to undertake the respective research [21]. This implementation demonstrates the capacity for many similar frameworks, such as Payback, the Balanced Scorecard, the Translational Research Organizations Performance Model, the Comprehensive Research Metrics model, the ITHS KLM–WHO model, the RE-AIM model and REF to develop this capability, given their shared characteristics [28, 42, 44, 45, 54, 55].

## Discussion

Health and medical research conducted under the umbrella of MRIs has a substantial impact beyond traditional academic outputs. RIAFs provide a mechanism to both demonstrate and encourage such impacts. This paper examined the objectives presented for research impact assessment in light of economic imperatives to justify on-going government support for HMR investment, leverage productivity via commercialisation and outcome-efficiency gains in the health system, and embed translation and impact into the research process.

The research found that many frameworks do not have an explicit purpose, nor are they systematically designed to realise these imperatives or other objectives, despite the relevance of purpose to their form, method and content. A potential reason for this omission lies in the hegemony of the ‘4A’ objectives of accountability, advocacy, analysis and allocation, presented in formative work by the RAND Corporation [38, 56]. Whether these objectives are explicit or implicit, the majority of the evaluated frameworks possess the capability to provide information for accountability, advocacy and strategic management decisions, i.e. analysis and allocation, to varying degrees. Collectively, they provide sufficient information to address the initial imperative to justify investment and provide for gradual improvement. This research sought to question whether the objectives included in ‘4A’ are sufficient to address the other imperatives, and specifically whether the objectives can be expanded to pro-actively encourage research impact in line with the latter two imperatives.

To the extent that Management Learning and Feedback seeks to inform upon the allocation of funds, such as the United Kingdom REF [35] and MCRI’s Research Performance Evaluation Framework [32], or the refinement of subsequent research activity, then most frameworks are indirectly concerned with accelerating translation and improving the productive impact of HMR. The main challenge with retrospective reviews relates to the time lag in these decisions. Retrospective reviews of research projects, programs or grants, such as the United Kingdom REF process typically take 12 months [35], if not years, for the feedback to actively inform research decisions. For regular evaluations, it is possible that expectations of the review will prospectively shape practice and consequently accelerate translation. However, the speed of translation will only be optimised if decisions to improve research translation and impact are made prospectively; that is, during the research process. When reviewed against the criteria for prospective implementation or speed of translation, many of the frameworks were either insufficient to meet this requirement or, while capable of adaption, were not designed for this purpose. Even where a pro-active objective is noted, other priorities can dilute the effect. For example, the United Kingdom’s REF [28, 35] sought to encourage research impact, but the adopted method (peer-reviewed case studies) was ultimately informed by pragmatic considerations of assessment across multiple disciplines, rather than the need to drive productive outcomes.

The potential implications for the development of new or evolved RIAFs for MRIs are numerous. First, the primary objective/s for a framework should be made explicit. Second, the framework should be designed to realise these objectives. Third, the qualities of existing frameworks inform upon the guiding principles necessary to realise different objectives. If the objective is to encourage productive research outcomes through greater integration of translation and impact considerations into the research process, then prospective implementation and a focus upon the speed of translation merits prioritisation [57].

The limitations of this study include the scoping literature review methodology, which may not have captured all potentially relevant studies. Inclusion of the knowledge exchange/translation literature and hand-searching of references should have ensured that the majority of key documents were captured. The focus upon frameworks applied to HMR may also have excluded relevant research with implications for the generalisation of the conclusions beyond health research. The main limitation relates to the evaluation. While mitigated by the independent review process, the determination of the criteria and the evaluation of frameworks according to these criteria introduces subjectivity as to the capabilities of each RIAF. However, modest subjective differences in categorisation should not act to undermine the over-arching conclusion regarding the disconnection between the capabilities of most existing RIAFs and the economic imperatives driving their development and implementation. Inclusion of a third reviewer may have strengthened the research design, but was precluded by resource constraints.

These issues raise a number of additional questions. If the objective is to optimise research translation and impact, consistent with the economic imperatives to drive productive gains, then where should a framework be implemented? At one or more points through the research process or at the institutional level? Through academic track records or the research process? How would such a framework fit with the peer-review process? The value of a RIAF integrated with the research process would be that it acts less as an additional administrative burden upon researchers, and more as an enabling mechanism for researchers to demonstrate and optimise the translation and impact of their work. What would the attitude of researchers be to such an approach? Is it viable in practice? Questions of to whom, within what timeframe, of causality and attribution, also remain a challenge [5, 28, 40, 44]. How would a framework address these issues? If the primary challenge is to change research culture, is a resolution to these issues necessary or merited? What does this imply for the form and positioning of a framework? Are motivations and incentives correctly aligned to realise the intended objectives? How would a more comprehensive researcher-focussed framework fit with national engagement and impact assessment frameworks, such as the United Kingdom REF [28] or the framework under development by the Australian Research Council [30]? This research represents the first in a suite of research papers that seek to define a draft RIAF for Australia’s MRIs. These questions need to be addressed in subsequent research.

## Conclusions

Significant expenditure has been incurred developing RIAFs, both in Australia and internationally. Greater resources will be consumed conducting research translation and impact assessments using such frameworks. Consequently, it is vital that these frameworks have the capacity to deliver upon their intended purpose.

This study demonstrates that, despite the centrality of purpose to evaluation, insufficient attention has been placed upon the question of ‘why?’. The main objective/s for a framework should be explicit and assessment frameworks should be explicitly designed to realise these objectives. The majority of RIAFs meet the objectives to provide accountability, and to varying degrees, advocacy and management learning and feedback. This capacity is adequate to address the first economic imperative to justify investment in HMR, but insufficient to optimise the actions, outcomes and impacts demanded by the second (leverage productivity) and third (quality improvement) economic imperatives. To address these imperatives, the capacity for a framework to be implemented prospectively and a focus upon the speed of translation merits prioritisation.

## Footnotes

1 At the time of writing, the Australian Research Council were undertaking a consultation process to develop an explicit Engagement and Impact Assessment framework that will provide more comprehensive information than contained with the existing Excellence in Research Australia, which is primarily a research quality framework.

2 Measurement of Research Impact and Achievement was piloted, but not fully implemented, by the Australian National Health & Medical Research Council.
